# Aqueous Extracts of Some Medicinal Plants are as Toxic as Lmidacloprid to the Sweet Potato Whitefly, *Bemisia tabaci*

**DOI:** 10.1673/031.009.1501

**Published:** 2009-05-05

**Authors:** Mazen A. Ateyyat, Mohammad Al-Mazra'awi, Talal Abu-Rjai, Mohamad A. Shatnawi

**Affiliations:** ^1^ Ash-Shoubak University College, Al-Balqa' Applied University, 17119 Al Salt, Jordan; ^2^ Faculty of Agricultural Technology, AI-Balqa' Applied University, 17119 Al Salt, Jordan; ^3^ Department of Pharmaceutical Sciences, Faculty of Pharmacy, University of Jordan, Amman, Jordan

**Keywords:** Biopesticides, botanicals, *Achillea* biebersteinii, *Artemisia inculta*, *Ballota undulata*, *Euphorbia hierosolymitana*, *Calium longifolium*, *Lepidium sativum*, *Pimpinella* anisum, *Phlomis syriaca* and *Retama raetam*

## Abstract

Aqueous extracts of nine plants, known to have medicinal activity, were tested for their toxicity against the sweet potato whitefly, *Bemisia tabaci* Genn. (Homoptera: Aleurodidae) compared to the toxicity of the insecticide, Imidacloprid. Extracts *of Lepidiuim sativum* L. (Brassicales: Brassicaceae) killed 71 % of early stage nymphs, which was not significantly different from mortality caused by Imidacloprid. Treatment of pupae with three plant extracts, *L. sativum, Achillea biebersteinii* L. (Asterales: Asteraceae), or *Retama raetam* (Forssk.) Webb and Berthel (Fabales: Fabaceae) prevented adult development, and treatment with *R. raetam* extract killed adults, at levels that were not significantly different from Imidacloprid. None of the other plants showed significant toxicity. However extracts of four plants, *Pimpinella anisum* L. (Apiales: Apiaceae), *Galium longifolium* (Sibth. and SM.) (Gentianales: Rubiaceae), *R. raetam* and *Ballota undulata* Bentham (Lamiales: Lamiaceae) had a repellent effect.

## Introduction

Plants may provide an alternative to currently used pesticides for the control of plant pests, as they constitute a rich source of bioactive chemicals (Kim et al. 2005; Daoubi et al. 2005). Recent studies have demonstrated the insecticidal properties of chemicals derived from plants that are active against specific target species, biodegradable to non toxic products and potentially suitable for use in integrated management programs (Markouk et al. 2000; Tare et al. 2004).

The sweet potato whitefly, *Bemisia tabaci* Gen. (Homoptera: Aleurodidae), is a key pest of vegetables in Jordan (Al-Musa et al. 1987). It is also a serious economic pest of agronomic, horticultural, and ornamental crops throughout warm regions of the world (Byrne et al. 1990; Brown 1994).

In the present study, the toxicity and repellency of aqueous extracts of nine plants known to have medicinal activity, were investigated against the sweet potato white-fly, *B. tabaci.*

## Materials and Methods

### Plant material

Nine plants known to have medicinal activity, *Achillea biebersteinii* L. (Asterales: Asteraceae), *Artemisia inculta* Del. (Asterales: Asteraceae), *Ballota undulata* Benth. (Lamiales: Lamiaceae), *Euphorbia hiersolymitana* Boiss. (Malpighiales: Euphorbbiaceae), *Galium longifolium* (Sibth. and SM.) (Gentianales: Rubiaceae), *Lepidium sativum* L. (Brassicales: Brassicaceae), *Pimpinella anisum* L. (Apiales: Apiaceae), *Phlomis syriaca* Boiss. (Lamiales: Lamiaceae) and *Retama raetam* (Forssk.) Webb and Berthel (Fabales: Fabaceae), were collected from their natural habitats, from different localities of Jordan. The identity of each plant species mentioned was verified and confirmed by Prof. Dawud M. Al-Eisawi (Department of Biology, Faculty of Science, University of Jordan) using live specimens and photographs. The parts of the different plants used in the experiments are given in Table 1.

**Table I.**
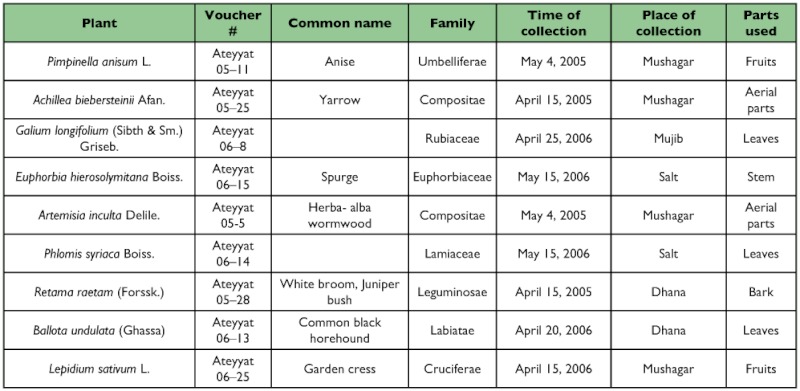
Voucher number, common name, family, time of collection, place of collection and partsused of the selected Jordanian medicinal plants.

### Preparation of the aqueous extracts

An aqueous extract was prepared by boiling 10 % wt/wt of the air-dried powdered plant part in sterile distilled water for 10 min and then cooled to room temperature overnight. The aqueous extracts were filtered using a Millipore filters (Millipore 0.2 mm, www.waters.com) to remove particulate matter. The final volume of each filtrate was completed to 100 ml with distilled water with 0.2% Tween 80 to account for the evaporated water during boiling. The aqueous extracts were prepared shortly before application. Negative controls represented by the distilled water contained the emulsifier Tween 80.

### Insecticide

The insecticide Imidacloprid (Confidor® 200SC, Bayer Crop Science, www.bayercropscience.com) was used as positive control treatment at the recommended field application rate of 0.25 ml/1.

### Insect culture

The colony of *B. tabaci* was maintained on tomato plants. To obtain immature whiteflies, 4-week-old greenhouse-grown tomato plants grown in a whitefly-free screened cage were trimmed to three fully expanded leaves and were transferred to the whitefly colony for 48 h. Adults were then aspirated from the plants, and the plants were placed in a separate cage. The synchronously-developing, uniformly-aged whitefly populations were then held until they developed to the appropriate stage.

### Egg mortality

Immediately after the adult whiteflies were aspirated from the plants, the plants were sprayed with extracts. Water or Imidacloprid were the negative and positive controls Initial number of eggs prior application ranged from 140 to 300 per plant. There were five replicates (plants) for each treatment. Eight days after treatment, the unhatched eggs and newly emerged nymphs were counted and the percent hatch calculated.

### Early stage nymphal mortality

Eight days after infestation, when first instar nymphs had emerged and attached to the leaf, the plants were sprayed as before. The number of first instar nymphs per plant ranged from 98 to 190. There were five replicates (plants) per treatment. Ten days after treatment, the number of dead nymphs were counted under a dissecting microscope. A nymph is considered dead if it was shrunken or its color changed. Normally developed nymphs to adult stage were also counted and the percentage of each was calculated.

### Late stage pupal mortality

Fourteen days after infestation, when most nymphs were in the red-eye stage, the plants were sprayed as before. The number of nymphs per plant ranged from 75 to 150. There were five replicates (plants). Seven days after treatment, when most of the pupae had emerged from control plants, the number of empty pupal cases and pupae that failed to emerge were counted and the percent of emergence was calculated.

### Adult mortality

A fully expanded leaf was placed in wet moss inside a Blackman box (Blackman 1971). The leaf was dipped into the solution of the required treatment and left overnight. About 30 adults were then introduced inside each box. Distilled water was used as negative treatment and imidacloprid was used as positive treatment. The number of dead whitefly adults was recorded after 48 hr. A whitefly adult was considered dead if it did not move after probing with a camel hair brush. Five replicates were made for each treatment.

### Repellency tests

Two fully expanded leaves of tomato were placed individually in vials containing water. One leaf was dipped in the tested plant extract and the other one was dipped in distilled water. The vials were placed in a plastic jar (20 cm diameter, 30 cm high) covered with fine netting material. About 50 immobilized adults were placed between the two vials. Numbers of adults attracted to each leaf was recorded after 3hr and 24hr. Five replicates were made for each treatment.

### Statistical analysis

Arcsin-transformed data were analyzed by ANOVA and means were separated by Tukey's Studentized Range test. Data of the repellency tests were analyzed using t-tests.

## Results

Over 80% of the eggs hatched and nymphs were able to emerge, regardless of treatment and all plant extracts showed no differences with both negative and positive controls (F = 2.62, df = 1O, 44; P = 0.0134) (Table 2). The highest percentage of dead early stage nymphs (71%) was caused by the extract of *L. sativum* that was not significantly different from the effect of Imidacloprid (F = 31.05, df = 10, 44; P = 0.0001) (Table 3). Extracts of three plants, *R. raetam, P. syriaca* and *A. inculata* were as toxic as the *L sativum* extract, but not as toxic as Imidacloprid.

**Table 2.**
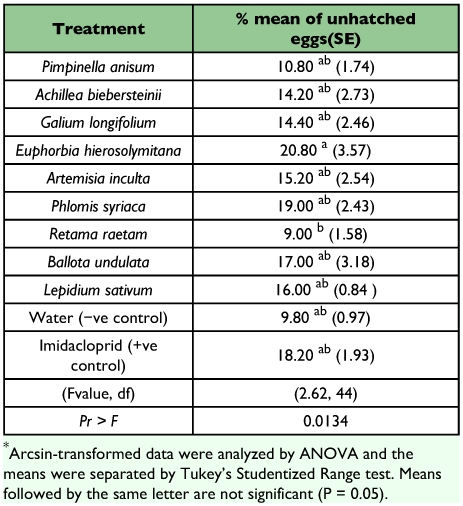
% means of unhatched eggs of Bemisia tabaci exposed to a number of plant extracts.

Treatment of pupae with three of the plant extracts, *L sativum, A. biebersteinii,* or *R. raetam* prevented adult development as well as Imiacloprid (F = 5.51, df 10, 44; P = 0.0001) (Table 4). Treatment with *R. raetam* extract killed adults was as effectively as Imiacloprid (F = 6.68, df 1, 44; P = 0.0001) (Table 5).

**Table 3.**
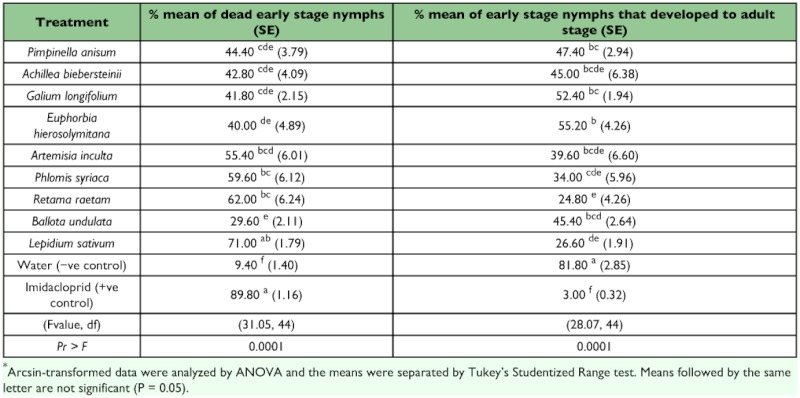
% means of dead early stage nymphs and *%* of early stage nymphs that developed to adult stage after exposure to a number of plant extracts.

**Table 4.**
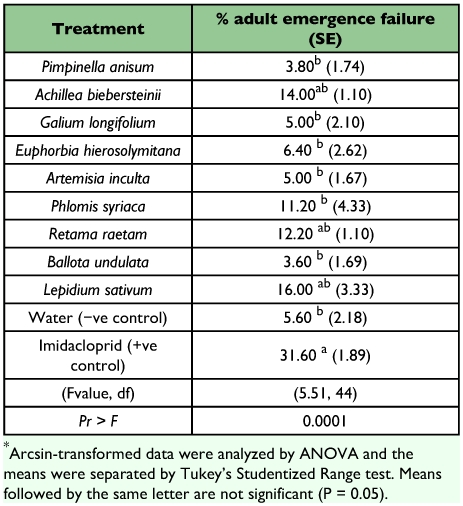
% adult eclosion failure after pupal exposure to a number of plant extracts.

**Table 5.**
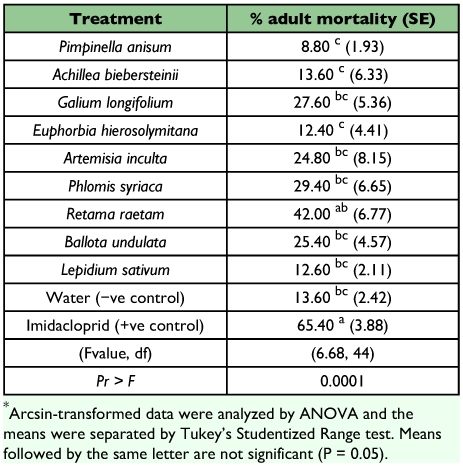
% mortality of adults exposed to a number of plant extracts.

**Table 6.**
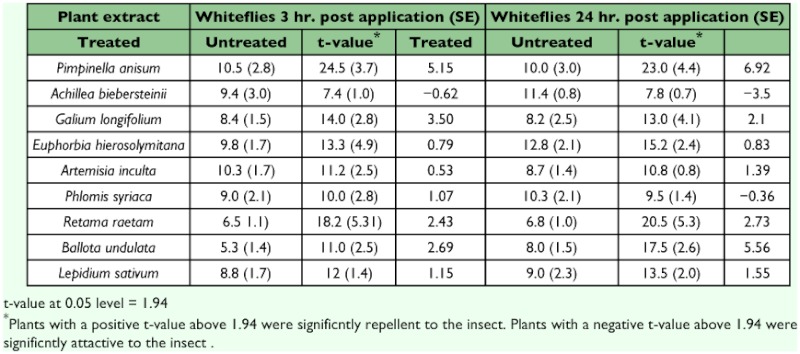
Average number of adults attracted to treated and untreated plants

The extracts of *P. anisum, G. longifolium, R. raetam* and *B. undulata* showed repellency effect to adults of whitefly compared with untreated plants (Table 6). However, leaves treated with extracts of *A. biebersteinii* were more attractive to the whitefly than untreated leaves by 24h after application.

## Discussion

Whitefly management has traditionally depended on the use of synthetic insecticides. However, the increasing resistance of *Bemisia* species to insecticides provides an impetus to use integrated pest control measures, including biopesticides and biological control to combat this pest. Biopesticides are based on natural products and synthetic analogs of naturally occurring biochemicals and are more acceptable than conventional pesticides because of their reputation for being less hazardous to humans and other non-target organisms (McCloskey et al. 1993). Among the biopesticides are chemicals derived from a variety of plant families. The biological activity of plant extracts against bacteria, fungi, viruses and insects has been reported (Bozsik 1996; Macedo et al. 1997; Unicini Manganelli et al. 2005).

In the present work, extract of *L. sativum* had toxicity that was not significantly different from the effect of Imidacloprid against early stage nymphs and pupae of *B. tabaci.* Treatment of pupae with three plant extracts, *L sativum, A. biebersteinii,* or *R. raetam* prevented adult development, and treatment with *R. raetam* extract killed adults, at levels that were not significantly different from Imiacloprid. *P. anisum, G. longifolium, R. raetam* and *B. undulata* had repellent effects on adults of *B. tabaci.*

*L sativum* belongs to Cruciferae family that contain glucosinolates (Burow et al. 2007). Glucosinolates are a class of thioglycosides found predominantly in plants of the order Brassicales. An anti-herbivore defense has been attributed to the products formed by myrosinase-catalyzed hydrolysis upon plant tissue damage (Burow et al. 2007). The leaves of *L sativum* are antiscorbutic, diuretic and stimulant (Uphof 1959; Chopra et al. 1986). The plant is administered in cases of asthma, cough with expectoration and bleeding piles (Chopra et al. 1986). The root is used in the treatment of secondary syphilis and tenesmus (Chopra et al. 1986). *A. biebersteinii* is rich in camphor, borneol and 1,8-cineole (Esmaeili et al. 2006). It is used as an antispasmotic, for abdominal pain and healing wounds. *R. raetam* contains flavinoids, has been used as an herbal remedy for diabetes and has been shown to have hypoglycemic activity in rats (Maghrani et al. 2005).

Repellent activity of some non-insecticidal agents could be attributed to the complex mixture of compounds that are detected by the susceptible insect (Schumutterer 1985). Anise is the common name of *P. anisum,* and is among the more ancient aromatic plants. The fruits of *P. anisum* are claimed to possess expectorant, stimulant, carminative, diuretic and diaphoretic properties. They are also used in flatulent colic and in some pharmaceutical preparations for asthma (Siddiqui et al. 2002). Phenylpropanoid derivatives from *Ballota nigra* exhibited a moderate antimicrobial activity against *Proteus mirabilis* and *Staphylococcus aureus* (Didry et al. 1999). Acetone extract of *Ballota hirsuta* leaves produced growth inhibition in larvae of the stored grain pest *Tribolium castaneum* (Passcual-Villalobos and Robledo 1999).

Considering toxic effects of *L sativum, A. biebersteinii,* and *R. raetam,* and the repellent effects of of *P. anisum, G. longifolium, R. raetam* and *B. undulata* against *B. tabaci,* it is possible that extracts of these plants can be used as natural control agents. Most of these plants are widely distributed and easy grown. Furthermore, the extraction method is simple and cost-effective and the application techniques could be relatively easily designed for on-farm use. Since *B. tabaci* transmits tomato leaf curl virus, developing new methods of control is obviously important.
